# Effects of Cleaning Protocols on Resin Bond Strength to Saliva-Contaminated Monolithic Zirconia

**DOI:** 10.3290/j.jad.c_2011

**Published:** 2025-04-30

**Authors:** Ozge Genc, Necla Demir, Mutlu Özcan

**Affiliations:** a Ozge Genc Graduate student, Graduate Prosthodontics, Department of Prosthetic Dentistry, School of Dentistry, University of Selcuk, Selcuklu, Konya. Design, and execution of experiments, data analysis, wrote and reviwed the manuscript.; b Necla Demir Associate Professor, Department of Prosthetic Dentistry, School of Dentistry, University of Selcuk, Selcuklu, Konya. Researched, designed, and conducted the study, wrote and reviewed the manuscript.; c Mutlu Özcan Professor, Clinic of Masticatory Disorders and Dental Biomaterials , Center for Dental Medicine, University of Zurich, Zurich, Switzerland. Design, and execution of experiments, data analysis, wrote and reviwed the manuscript.

**Keywords:** adhesion, dental materials, monolithic zirconia, pumice, resin bond strength, universal cleaning agent

## Abstract

**Purpose:**

Considering the significant weakening of the bonding of resin cements to saliva-contaminated zirconia restorations, this study aimed to investigate the effect of various surface treatment methods on the bonding of self-adhesive resin cement to zirconia after various cleansing methods.

**Material and Methods:**

A total of 105 monolithic zirconia specimens were cut from pre-sintered blocks using a water-cooled precision diamond saw. Specimens were kept in the artificial saliva except for the control group. The specimens were then cleaned using one of the following methods: 1. Air-water spray, 2. Isopropyl Alcohol, 3. Pumice, 4. Universal cleaning agent (Ivoclean), 5. Sandblasting, 6. Sandblasting + Ivoclean. Specimens were bonded to self-adhesive resin cement. Specimens were thermocycled for 5,000 cycles after cementation and tested in shear mode (1 mm/min). Images were obtained using a stereomicroscope, scanning electron microscope, and energy-dispersive spectroscopy. Data were analyzed using ANOVA, and multiple comparisons were performed with the Duncan test (α = 0.05).

**Results:**

The mean shear bond strength values were as follows in descending order: Sandblasting + Ivoclean (9.3 MPa) > sandblasting (8.59 MPa) > Ivoclean (7.21 MPa) > Pumice (4.82 MPa) > Air-water spray (4.15 MPa) > Control (3.65 MPa) > Isopropyl alcohol (3.04 MPa). Significant difference was observed between sandblasting and Ivoclean groups, and between sandblasting and sandblasting + Ivoclean groups (P < 0.05). A significant difference was also found between the Ivoclean and sandblasting + Ivoclean groups. The groups treated with sandblasting + Ivoclean, sandblasting, and Ivoclean showed a significant difference compared to all other surface treatment groups. There was no significant difference in shear bond strength among the control, air-water, alcohol, and pumice groups (P > 0.05). Sandblasting, Ivoclean, and Ivo-clean after sandblasting applications were found to deliver significantly higher (P < 0.05) adhesion compared with air-water, pumice, and alcohol applications.

**Conclusions:**

Subsequent applications of Ivoclean after sandblasting established a stronger bond between self-adhesive resin cement and monolithic zirconia than other cleaning methods tested.

**Clinical Implications:**

Following airborne particle abrasion, intaglio surfaces of zirconia restorations should best be cleaned using Ivoclean.

The use of zirconia ceramics in esthetic dentistry has increased rapidly due to their biocompatibility, esthetic, and mechanical properties.^3^ The most common clinical failure in zirconia-based restorations is fracture or chipping failures.^1^ Monolithic zirconia ceramics have been introduced recently to eliminate such clinical failures.^1^ Zirconia can also be used to produce the core and superstructure for implant or tooth-supported restorations.^1^ More recently, monolithic zirconia ceramics, which do not require layering with superstructure ceramics, have started to be widely used in dental practice.^1,38^ Monolithic CAD/CAM zirconia ceramics have some significant advantages, such as high material strength, conservative tooth preparation, pleasing esthetic appearance, saving time both in laboratory and clinical studies, and no chipping complications.^1,3^

Some mechanical and chemical surface treatments are applied to the ceramic surface to increase the bonding quality between the zirconia ceramic and the tooth structure.^4,7^ While micromechanical retentions can be achieved by increasing the surface area with sandblasting, long-term chemically durable bonding can be obtained using resin cement and/or ceramic primer containing phosphate monomer, specifically with MDP.^4,7,19^ 10-MDP binds to the hydroxyls on the zirconium oxide surface while it is chemically adsorbed.^4,7^

The acidic monomers in the structure of the MDP react with oxides within the zirconia surface, like the reaction between silane agents and silica-based ceramics.^41,65^ Organophosphate monomers also have an organofunctional part containing a methacrylate group that can co-polymerize with the monomers of composite resin systems and silane coupling agents.^30^ Therefore, using cement and primers containing MDP increases the bond strength between zirconia ceramics and the tooth structure.^60^ Moreover, while self-adhesive cements are designed to be used without requiring any extra physical or chemical preparation of the substrate, some include application of a separate ceramic primer that contains MDP.^40^

The contamination of dental restorations with saliva, blood, and silicone markers is inevitable during clinical procedures.^13, 15,18,20^ The boundary strength of interfaces between the restorations and the teeth probably decreases due to exposing both organic and inorganic contaminants in saliva.^3,5,18,27,31^ Accordingly, mechanical and chemical cleaning methods are used to clean the contaminants and increase the bond strength between the constituents. The most common chemical cleaning agents are alcohol (70–96% isopropanol),^13,45,63^ acetone, phosphoric acid (35–37%),^63^ sodium hypochlorite, hydrogen peroxide, and sodium dodecyl sulfate.^30^ In general, the sandblasting process,^24,30,63^ is still accepted as one of the most effective^36^ mechanical cleaning methods. A universal cleaning agent (IC) (Ivoclean, Ivoclar Vivadent) contains sodium hydroxide and zirconium oxide particles, which have been proposed to clean the zirconia surface by absorbing the phosphate contaminants from the contaminated zirconia surface.^31^ In previous *in vitro* studies,^20,24,30,63,64^ IC was preferred on zirconia surfaces treated with sandblasting.

This study was planned to examine the increased cleansing effect of IC on the bond strength of contaminated zirconia samples with and without sandblasting treatment. To the best of our knowledge, there is a lack of investigation concerning the cleaning ability of IC used on zirconia surfaces without pre-sandblasting, and there is no study directly related to the cleaning capability of pumice application on monolithic zirconia surfaces. Kwak et al’s study only suggests that a simple surface cleaning of the zirconia glaze layer with a prophy cup and pumice followed by silane application can be sufficient for bonding to orthodontic brackets, being safer than the latter. It is also logical to consider alternative cleaning methods that provide a similar level of effectiveness as air abrasion.^14^ The novelty of the study is to reveal the cleansing effects of IC agent alone and pumice application on saliva-contaminated monolithic zirconia surface.

Therefore, this study aimed to investigate the effects of artificial saliva contamination and different cleaning methods on the shear bond strength of monolithic zirconia ceramics. The null hypothesis was that there would be no difference in cleaning methods regarding bond strength between the resin cement and saliva-contaminated zirconia.

## MATERIAL AND METHODS

A total of 105 zirconia (In Coris TZI, Sirona Dental, Benscheim, Germany) specimens (15×12×2 mm^3^) were cut from pre-sintered blocks using a water-cooled precision diamond saw (Isomet 1000, Buehler, Lake Bluff, IL USA). Based on the results obtained from the power analysis using G*Power software version 3.1.10, a minimum of six specimens per group was necessary to achieve 95% confidence (1-α), 97.8% test power (1-β), and an effect size of f = 0.885. Accordingly, ten specimens were prepared for each subgroup. The specimens were then randomly distributed into seven groups, including a control group. The materials used in this study are shown in Table 1. These specimens were then sintered at 1510°C according to the manufacturer’s instructions in a high-temperature furnace (Protherm, Ankara, Turkey), and they were polished with silicon carbide paper (no. 600, Struers, Copenhagen, Denmark) under copious water by a single trained operator. After that, specimens were cleaned ultrasonically in distilled water for 10 min and dried with oil-free air.

**Table 1:** Materials, compositions, manufacturers, and batch numbers

	Material type	Composition of the material	Manufacturer	Batch number
In Coris TZI	Pre-sintered Y-TZP monolithic zirconia block	ZrO_2_, HfO_2_, Y_2_O_3_, Al_2_O_3_, Fe2O3 and other oxides	Sirona Dental, Benscheim, Germany	2016279863
Clearfil Ceramic Primer Plus	Universal Primer	MDP, g-MPTS, ethanol	Kuraray, Tokyo, Japan	3P0053
Panavia SA Cement Plus Automix	Adhesive resin cement	Bis-GMA, TEGDMA, MDP, hydrophobic aromatic dimethacrylate, HEMA, hydrophobic aliphatic dimethacrylate, silanized Ba glass, silanized colloidal silica, sodium flour, CQ, peroxide, catalysts, accelarators, pigments	Kuraray, Tokyo, Japan	770066
Imıpomza	Surface cleaning agent	Pumice	Imıcryl, Konya, Turkey	21C766
Alcohol	Surface cleaning agent	96% isopropyl alcohol C_3_H_8_O	Selcuk University Science Faculty Biochemistry Lab, Konya, Turkey	
Ivoclean	Surface cleaning agent	Sodium hydroxide, ZrO_2_, water, polyethylene glycol, pigments	Ivoclar Vivadent, Schaan, Liechtenstein	X16434
Al_2_O_3_	Surface cleaning agent	99.5% Aluminum oxide	Renfert, Hilzingen, Germany	2177995


**Fig 1:**
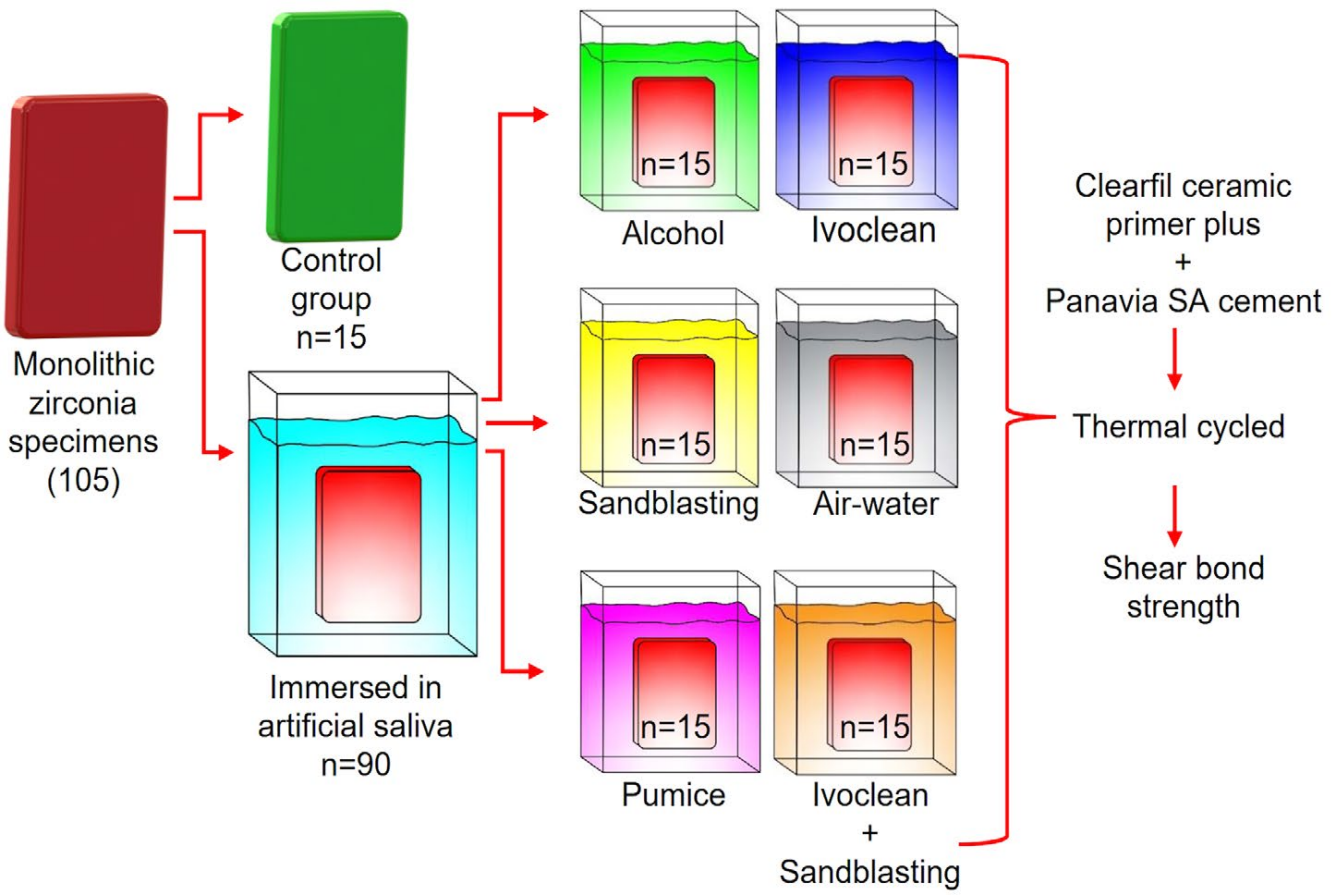
The contamination and cleaning procedures for each group.

With the exception of the specimens in the control group, all other specimens were submerged in artificial saliva (Table 2) for a duration of 1 min at a temperature of 37°C. The control group did not undergo neither sandblasting nor saliva contamination. Following contamination with artificial saliva, the specimens were subsequently cleansed using one of the provided cleaning techniques: 1. An air-water spray (AW) is used for a duration of 15 s, followed by drying it with oil-free air for another 15 s. 2. Isopropyl alcohol (AL) (Selcuk University Faculty of Science, Biochemistry Laboratory): submerge in a solution of 96% isopropanol for a duration of 1 min, followed by rinsing with water spray for 15 s and drying with oil-free air for 15 s. 3. Pumice (P): pumice (Imipomza, Imicrly, Turkey ) water slurry with rotating prophy brush cup is used to clean the surface for a duration of 30 s, followed by rinsing with a water spray for 15 s and drying with oil-free air for 15 s. 4. The surface was treated with Ivoclean (IC) (Ivoclar, Ivoclar Vivadent, Schaan, Liechtenstein) agent for a duration of 20 s. It was then rinsed with water spray for 15 s and dried using oil-free air for 15 s, following the guidelines provided by the manufacturer. 5. Sandblasting (SB): air-abraded with 50 Al_2_O_3_ (Renfert, Hilzingen, Germany) at 0.25 MPa for 20 s at 10 mm, then rinsed with water spray for 30 s and dried using oil-free air for 10 s. 6. Sandblasting + Ivoclean (SI): air-abraded with 50 Al_2_O_3_ (Renfert, Hilzingen, Germany) at 0.25 MPa for 20 s at 10 mm, then rinsed with water spray for 30 s and dried with oil-free air for 10 s. After this process, the specimens were treated with Ivoclean (Ivoclar, Ivoclar Vivadent, Schaan, Liechtenstein) for 20 s, and rinsed with waterspray for 15 s and dried with oil-free air for 15 s. The contamination and cleaning protocols for each group are outlined in Figure 1.

**Table 2:** Chemical composition of artificial saliva^47^


2000 mg/L C_8_H_8_O_3_
10,000 mg/L Na CMC (C_8_H_15_NaO_8_)
58.87 mg/L MgCl_2_ . 6H_2_O
166.11 mg/L CaCl_2_ . 2H_2_O
417.6 mg/L K_2_HPO_4_
624.31 mg/L KCl
0.05 mg/L F


Ceramic primer (Clearfil Ceramic Primer Plus; Kuraray, Noritake Dental, Tokyo, Japan) was then applied on the monolithic zirconia surface with a microbrush for 10 s and air dried for 5 s before cementation after the cleaning procedures. A Teflon mold (6 mm × 3 mm) was used to demarcate the bonding area on the zirconia surface of each specimen. The self-adhesive resin cement (Panavia SA Cement Plus Automix, Kuraray, Noritake Dental, Tokyo, Japan) was applied into the Teflon mold and photo-polymerized with an LED light-curing unit (VALO, Ultradent, South Jordan, UT, USA) at standard mode (1000 mW/cm^2^) 60 s in total. Prior to debonding, all specimens were kept in at 37°C distilled water in an incubator (Nüve Incubator EN 120, NÜVE, Turkey) for 24 h and then subjected to 5000 thermocycles between 5°C and 55°C water baths with a dwell time of 30 s and transfer time of 5 s between each bath.

**Figs 2a to g:**
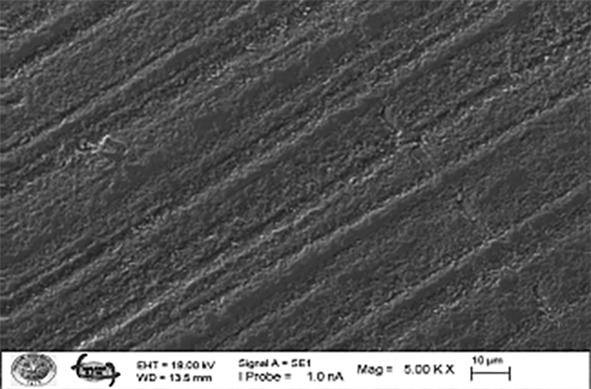

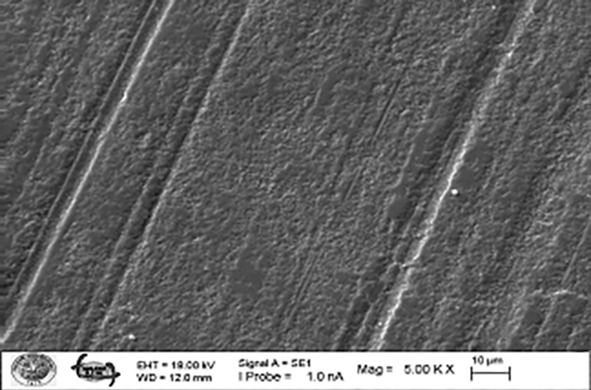

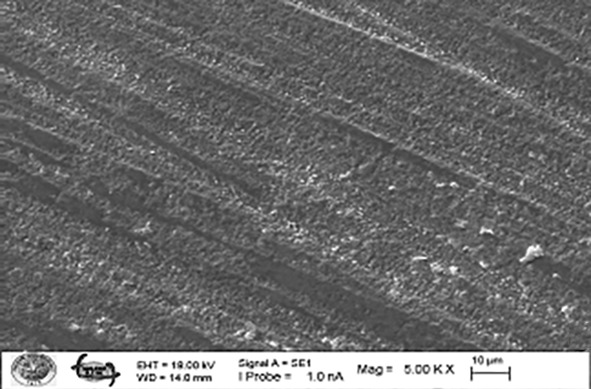

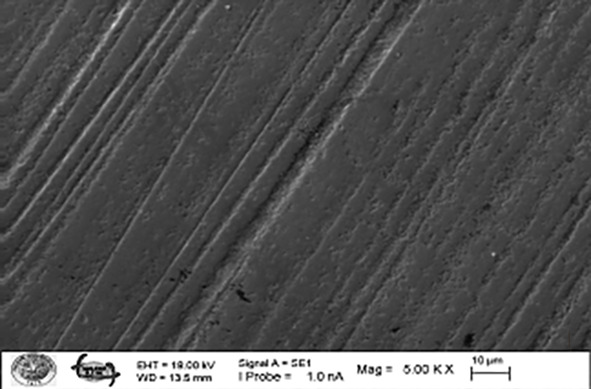

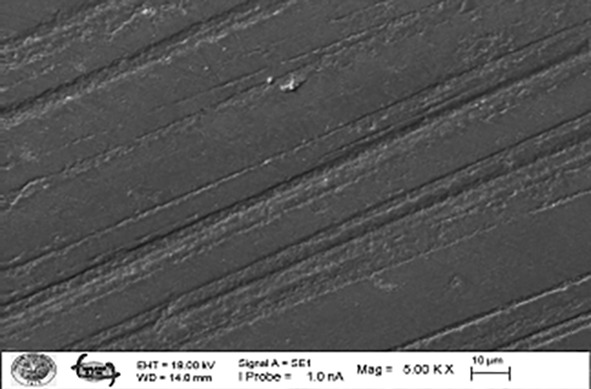

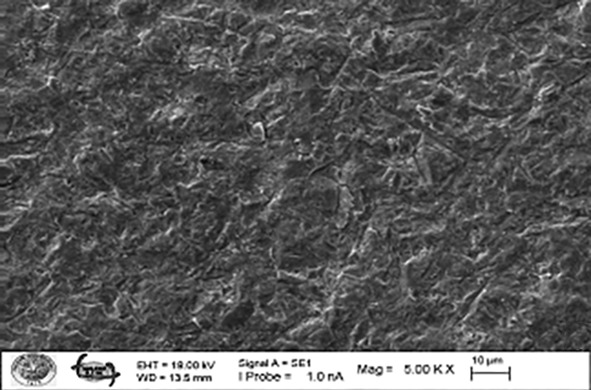

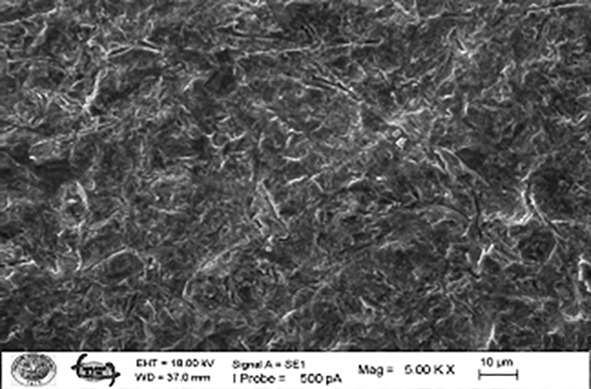
Representative SEM micrographs of the surface-treated zirconia after saliva contamination (x5000). (a) Control group; (b) zirconia surface after cleaned with alcohol; (c) zirconia surface after cleaned with air-water; (d) zirconia surface after cleaned with pumice; (e) zirconia surface after cleaned with Ivoclean; (f) zirconia surface after sandblasting; (g) zirconia surface after sandblasting + Ivoclean.

All specimens were tested in a shear mode using a shear testing apparatus in a universal testing machine (DVT Devotrans GP, Istanbul, Turkey) at a 0.5 mm/min crosshead speed. A chisel-shaped tip was used in the shear bond strength test to apply force between the substrate and the resin, which was positioned as close as possible to the bonding surface. According to ISO TR 11405, the load cell should operate at a speed ranging from 0.45 to 1.05 mm/min.^6^ The shear bond strength values (MPa) were calculated by dividing the peak load at the failure by the specimen surface area (Fig 2).

After the shear bond strength tests, the specimens were examined under an optical stereomicroscope (SZ-PTOlympus, Tokyo, Japan) at a magnification of ×20 to define the failure types. The failure modes were classified as follows: Adhesive failure: The failure occurs between the zirconia and the resin cement; Cohesive failure: The failure that occurs in the resin cement; Mixed failure: A combination of these failure modes.

After saliva contamination and shear bond strength test, one specimen from the control and each surface treatment group was examined using a scanning electron microscope (SEM) (Carl Zeiss Evo LS10, Oberkochen, Germany). Scanning electron microscopy examinations were carried out at 80×, 500×, 1.00 KX, and 5.00 KX magnifications.

Micro-analyses of the treated surfaces were conducted using an energy dispersive spectrometer (EDS) (Carl Zeiss Evo LS10, Oberkochen, Germany), at random areas on the specimens for elemental analysis. The shear bond strength values between the monolithic zirconia specimens and self-adhesive resin cement were statistically analyzed with a computer software (IBM SPSS V23 (IBM SPSS Statistics, v23; IBM). The data were normally distributed and therefore one-way analysis of variance was used to compare the bond strength values according to the groups. Multiple comparisons were performed with the Duncan test. Significance level was taken as P < 0.05 in all tests.

## RESULTS

**Figs 3a to g:**
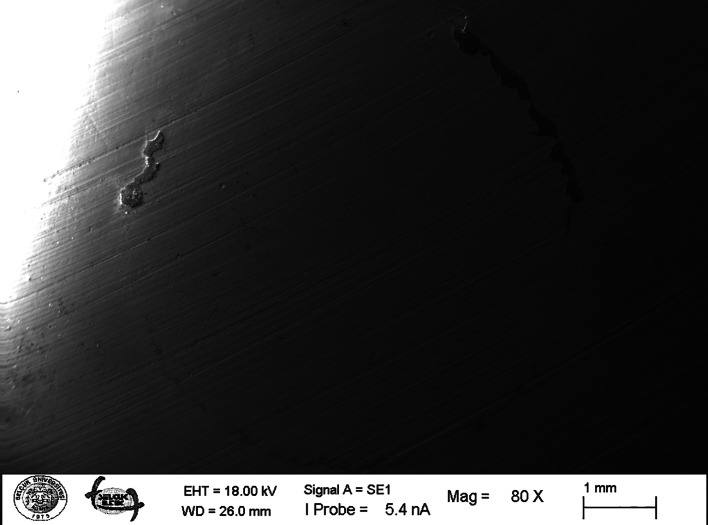

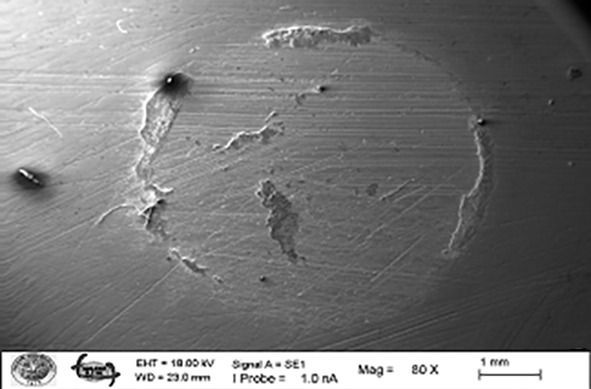

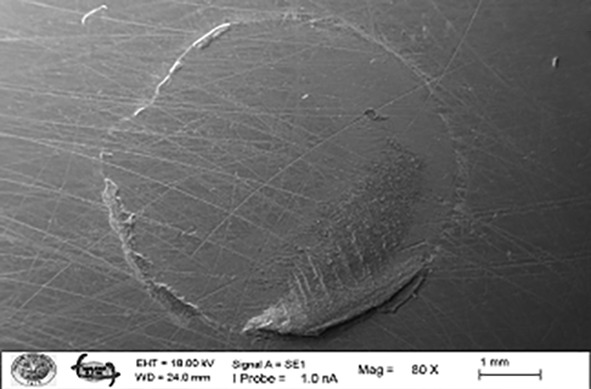

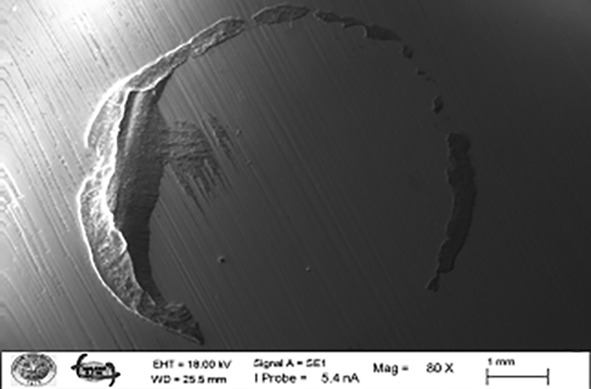

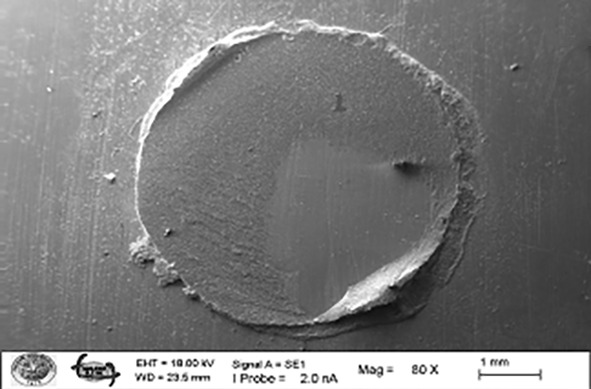

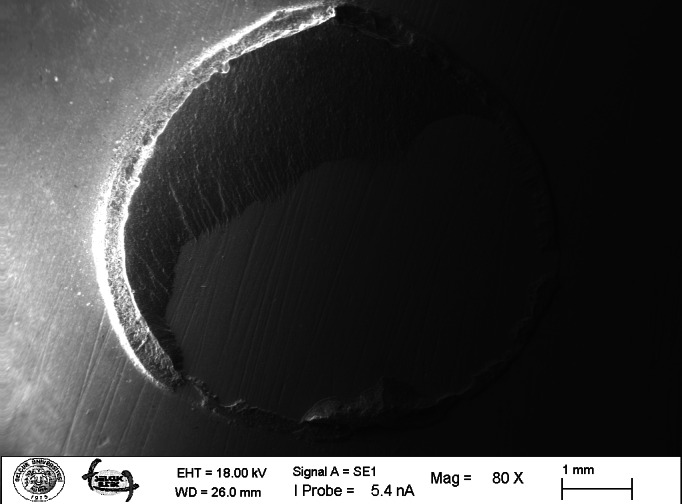

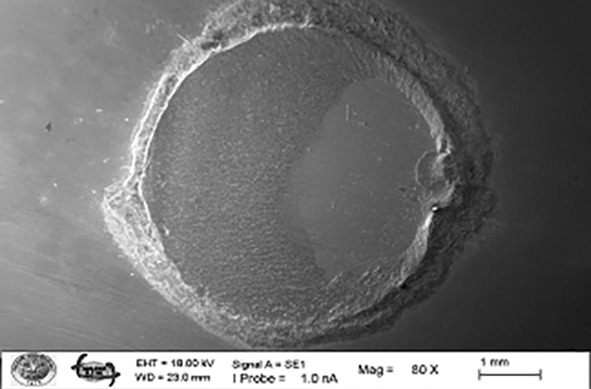
Representative SEM micrographs of the debonded zirconia surfaces (x80) (a to d) The failure mode was classified as adhesive for control; (e to g) The failure mode was classified as mixed for Ivoclean, sandblasting, and sandblasting + Ivoclean group.

Descriptive statistical values are presented in Table 3. The mean shear bond strength values significantly differed according to the groups (P < 0.001). According to the results, the group of sandblasting and Ivoclean showed the highest mean shear bond strength value, while the group of isopropyl alcohol showed the lowest. The results of the study indicated statistically significant differences in shear bond strength between the sandblasting + Ivoclean, sandblasting, and Ivoclean groups. In contrast, the Control and Alcohol groups exhibited similar shear bond strength values. The Air-water and Pumice groups also showed similar shear bond strength values. No significant differences were observed between the control, air-water, alcohol, and pumice groups. However, the sandblasting + Ivoclean, sandblasting, and Ivoclean groups demonstrated statistically significant differences compared to the control, air-water, alcohol, and pumice groups. Failure analysis revealed mixed failure modes in all specimens treated with Ivoclean, sandblasting, and sandblasting + Ivoclean. In contrast, adhesive failures were observed in the control, air-water, alcohol, and pumice groups. No cohesive failure was noted in any groups after contamination with artificial saliva. A statistically significant correlation was obtained between the groups and failure types (P < 0.001). While 100% of the Ivoclean group, sandblasting, sandblasting + Ivoclean groups had mixed type, 53.3% of the alcohol and air + water groups showed mixed type, 66.7% of the pumice group, and 60% of the control group had mixed type of failures (Table 4). SEM images of the surfaces of the specimens were also examined after surface treatments (Figs 2a to g) and following shear bond strength test at a magnification of 80×, 500×, and 1.00 KX, 5.00 KX (Figs 3a to g). Figures 2a to g display the SEM with a 5000 magnification level of seven groups for zirconia surface morphology after different cleaning methods, wherein morphological changes can be seen among the cleaning method groups, without contamination or cleaning. Figure 2a displays the grains on the zirconia surface in an uncontaminated state. Roughness caused by polishing with silicon carbide paper can be seen on the non-sandblasted surface. In Figure 2b, the zirconia surface is shown post-contamination with saliva, revealing the presence of small particles distributed across the zirconium surface after alcohol treatment. Roughness caused by polishing with silicon carbide paper can be seen on the non-sandblasted surface. In Figure 2c, it was seen that there were artificial saliva residues on the sample surface after air-water spray. Roughness caused by polishing with silicon carbide can be seen on the surface without sandblasting. In Figure 2d, it was seen that there was a large amount of artificial saliva residues on the sample surface after pumice cleaning, which was also supported by our EDS results. Roughness caused by polishing with silicon carbide can be seen on the non-sandblasted surface. A small amount of artificial saliva contaminants remained on the surface after cleaning with Ivoclean, which was also supported by our EDS results (Fig 2e). In Figure 2f, it was observed that the surface was quite rough after sandblasting. The sandblasted surfaces were very rough, and the image at 5.00 KX showed that artificial saliva contaminants were present between the microporosities in accordance with the EDS results (Figs 2f, g).

**Table 3:** Descriptive statistics of shear bond strength of all test groups

	(MPa)
Mean ± SD	Min–Max
Control	3.65 ± 1.20^ab^	1.88–6.95
Air-water	4.15 ± 1.08^bc^	2.44–5.86
Alcohol	3.04 ± 0.93^a^	1.43–4.63
Pumice	4.82 ± 1.19^c^	3.20–7.79
Ivoclean	7.21 ± 1.56^d^	4.38–9.88
Sandblasting	8.59 ± 1.31^e^	6.85–11.05
Sandblasting + Ivoclean	9.73 ± 2.23^f^	6.26–13.90
Means with same lowercase letters (a to f) are not statistically different.

**Table 4:** The frequency of the failure modes of the test groups

	Adhesive	Mixed	P
Ivoclean	0 (0)	15 (100)	< 0.001
Sandblasting	0 (0)	15 (100)
Sandblasting+Ivoclean	0 (0)	15 (100)
Alcohol	7 (46.7)	8 (53.3)
Air-water	7 (46.7)	8 (53.3)
Pumice	5 (33.3)	10 (66.7)
Control	6 (40)	9 (60)
*Fisher–Freeman-Halton test, frequency (percentage)

Saliva contaminants were observed in the microporosities of specimens for all surface treatment groups, supported by EDS analysis. According to the EDS analysis, the order of the groups according to the percentage of carbon on the surface was as follows: Ivoclean (0.1) < Sandblasting (0.49) < Sandblasting + Ivoclean (0.59) < Air-water (0.78) < Pumice (0.92) < Isopropyl alcohol (2.9) (Table 5), indicating that organic substance from saliva remained on the zirconia surface and could not be eliminated entirely, even when cleansing agents were applied. In addition, the EDS investigation revealed that the zirconia surface primarily consisted of zirconium and oxygen (Table 5). However, the presence of carbon, derived from saliva composition, decreased in the IC group, followed by the sandblasting and sandblasting + IC groups.

**Table 5:** The percentage of elements from EDS analysis

Groups	Weight %
Zr	O	C	Si
Control	73.45	24.01	0.0	2.07
Air-water	74.00	22.76	0.78	2.06
Alcohol	72.38	22.47	2.9	1.99
Pumice	74.21	22.27	0.92	2.06
Ivoclean	74.69	22.10	0.1	2.51
Sandblasting	71.63	23.13	0.49	1.87
Sandblasting+Ivoclean	71.19	24.37	0.59	1.03


## DISCUSSION

Contamination of zirconia restorations during the try-in procedure is unavoidable and can potentially weaken the adhesion of resin cement to zirconia.^10^ Previous studies have indicated that when the zirconia surface is contaminated with saliva, the phospholipids in the saliva can adhere to and occupy the outer oxide layer of the zirconia. This reduces the amount of the oxide layer available for bonding with 10-MDP. Consequently, if the contaminant is not properly removed, the bond strength of the zirconia restoration may be compromised.^4,25^ The present study examined the saliva contamination and the cleaning effect of various surface treatments on the bonding performance of monolithic zirconia ceramics. The null hypothesis that there would be no difference between the cleansing agents in bonding effectiveness was partially rejected. However, using human saliva in experimental studies may lead to ethical concerns or problems in reproducibility and standardization of experiments due to human variation.^22^ Therefore, artificial saliva was preferred in this study to standardize the experimental conditions.

One of the cleaning methods used to clean contaminants in the study is 96% isopropyl alcohol. Since alcohol causes cell lysis and protein denaturation, it has been used in various studies for surface cleaning.^13,32,45,47^ In this study, isopropyl alcohol-applied specimens showed the lowest shear bond strength value with 3.04 MPa. In previous studies, specimens in the alcohol group showed lower bond strength values than specimens in the air-water group, which is similar to this study.^9,18,62^ The low bond strength values observed in alcohol-treated samples can be explained by the fact that the applied alcohol cannot completely remove the organic material accumulated on the zirconia surface. In addition, alcohol residues remaining on the surface may react chemically with the phosphate monomers in the MDP structure and prevent the formation of a higher bond strength. Therefore, this chemical interaction may obtain low bond strength values in alcohol-applied samples.^9,18,62^

It has been stated in various studies that saliva contaminants on the zirconia surface cannot be sufficiently removed by applying air-water spray.^3,13,53,55^ After saliva contamination, non-covalent adsorption of salivary proteins occurs on the surface of restorative materials. It is not possible to remove this organic coating with water.^37,62^ This study hypothesizes that using the active water spray procedure increases the kinetic energy at the surface and thus reduces saliva contamination more effectively, but insignificantly, compared to the control group. Additionally, the higher tensile bond strength observed in the samples treated with air-water spray compared to the control group might be attributed to the use of artificial saliva in the cleaning process. Also, in this study, the specimens were not sandblasted before being contaminated with artificial saliva. In other studies, sandblasting before contamination may have caused greater penetration of saliva contaminants by causing roughness on the surface^18,41,62^ where water cleaning typically resulted in lower adhesion.^4,35,62^ This situation raises questions about the effectiveness of the cleaning methods.^64^ Additional particle abrasion could potentially compromise long-term durability.^29^ In this study, pumice in slurry form with a rotating prophy brush cup, commonly used for tooth and composite surfaces,^56^ was applied as a new cleaning method for the intaglio surface of ceramics. In a study by Kwak et al,^28^ the authors recommended a simple surface cleaning of the zirconia glaze layer with a prophy cup and pumice before bonding orthodontic brackets. Therefore, as the findings of this study show, pumice did not increase the shear bond strength of the brackets.^28^ The shear bond strength values ​​suggested that pumice application showed insignificantly better values than air-water spray to remove saliva contaminants from zirconia ceramics. Various studies have shown that pumice application causes roughness on the surface.^44,49^ In this study, the pumice application showed higher bond strength than the air-water spray group, possibly because it caused roughness on the ceramic surface. As a novelty of our study, when previous studies were examined, no research or clinical study was found in which pumice was used to clean saliva contaminants from monolithic zirconia surfaces. Further studies should validate this finding.

When the surfaces were cleaned with isopropyl alcohol, air-water spray, and pumice, shear bond strength was restored and did not differ significantly from uncontaminated surfaces. These findings agree with several previous studies showing the effectiveness of these cleaning agents in removing organic matter from saliva-contaminated surfaces.^57^

Air abrasion has been identified as an effective method to improve the bond strength of resin to contaminated zirconia surfaces.^14^ To maximize the effectiveness of cleaning pastes for resin bonding, it is recommended to follow up with another effective cleaning method after using this technique.^14^ This study safely performed sandblasting with 50 µm Al_2_O_3_ to remove artificial saliva contaminants, consistent with previous studies.^33,52^ Unlike prior research, not all samples were sandblasted before contamination; sandblasting was used as both a surface treatment and a method to remove contaminants after artificial saliva contamination. According to the studies, the bending strength of 3Y-TZP may increase^11,48^ or decrease^11^ depending on the type, size, and air pressure of the abrasive particles used in the sandblasting process. For this reason, it may be recommended to use low-pressure sandblasting with a primer containing MDP or to completely eliminate the sandblasting process in order to reduce the negative effect of high-pressure sandblasting on the mechanical properties of the ceramic and to provide a strong bond with the resin cement.^61^ In line with this information, Clearfil Ceramic Primer Plus (Kuraray), a primer containing MDP, was used after applying low-pressure Al_2_O_3_ to zirconia samples. At the same time, the sandblasting process may remove the contaminants that will adversely affect the chemical bonding on the ceramic surface and create active zirconia surfaces that can bond with the resin cement containing phosphate monomers.^64^ Although zirconia surfaces are highly hydrophobic and have low surface energy, sandblasting increases the surface energy and provides micro retention. Sandblasted zirconia surfaces without saliva contamination can be considered relatively hydrophilic, thus achieving high bond strength values.^64^ In this study, the positive effect of sandblasting on bond strength may be due to this factor. However, excessive sandblasting may cause damage to the zirconia surface, deteriorating the flexural strength of monolithic zirconia.^26^ On the other hand, sandblasting devices are not available in every clinic, and clinicians do not prefer the sandblasting process because the pollution of the environment makes it challenging to perform sandblasting. Therefore, applying a cleaning solution to the bond surfaces of the restorations after contamination may be considered as a more appropriate cleaning method and strengthen the bonding performance.^24^ The application of sandblasting enhances the surface energy and facilitates micro retention.^64^ However, sandblasting can cause surface defects in zirconia, including flaws, plastic deformation, embedded abrasive alumina, and microcracks. These defects can negatively impact the mechanical properties of zirconia and reduce its fracture strength. Due to the stress-induced transformation of zirconia ceramics, the surface structure may change when subjected to air-particle abrasion, potentially affecting its long-term performance.^23,29^ Therefore, the pursuit of alternative techniques and a replacement for alumina air-particle abrasion, which can enhance the robustness and endurance of the resin-zirconia bonding interface without causing harm to the zirconia surface, has emerged as a formidable task. Nevertheless, air-particle abrasion methods alone are insufficient for achieving resin cement adhesion to zirconia ceramics.^23,29^ A study conducted by Ahmed et al^2^ showed that Monobond Plus has the ability to improve the durability of zirconia copings. This silane primer is preferred in this study as a pre-treatment method before resin cementation. According to Samran et al^47^ the functional phosphate monomers, specifically MDP, in the resin cement formed a durable chemical bond with ZrO_2_ particles in the universal cleaning agent compared to the functional phosphate monomers present in the other self-adhesive resin cement studied.^47^

In this study, ZrO_2_ particles containing a universal cleaning agent and 10-MDP containing resin cement combination might have also manifested in markedly higher bond strength to the zirconia surface. Non-abrasive cleaning solutions have been developed as an alternative method to decontaminate the bonding surfaces of prosthetic restorations following intraoral try-in procedures.^29^ Thus, employing a cleaning agent to treat the bond surfaces of ceramics following saliva contamination could be a more efficient approach to cleanse and enhance the cementation bond.^4^ In their study, Martinez et al^29^ also determined that using a cleaning paste was the most efficient approach for eliminating saliva contamination. The use of cleaning agents such as the universal solution IC on the inner surface of the restorations with the help of micro brushes can be considered.^3,24,63^ According to the scientific data shared by the manufacturer, the direction of the chemical reaction depends on the concentration of the substances to be reacted. Therefore, high-concentration material is more likely to react than low-concentration material. Ivoclean consists of an alkaline suspension of zirconium oxide particles. Phosphate contaminants on the ceramic surface will bind to IC due to the size and concentration of particles. In this way, the phosphate contaminants actively present on the zirconia surface are absorbed, and the zirconia surface gets cleaned.^31^ In previous studies, control and tested samples were pre-sandblasted before being contaminated with human saliva.^3,13,20,24^ Therefore, as another novelty of this study, no studies show the effect of IC and other surface treatments individually compared with sandblasting to remove saliva contaminants and bond strength. Saliva may interfere with the bond strength by adhering to the surface of lithium disilicate restorations, which reduces the surface of the restoration to be wetted and lowers its surface-free energy.^51^ The primary mechanism in alkaline-based groups is their alkalinity, which effectively eliminates any remaining organic contaminants. Ivoclean is a potent alkaline cleansing paste that includes zirconia particles. It was asserted to possess a significant attraction to the phosphate group, leading to the elimination of impurities in saliva. Therefore, alkalinity served as the primary mechanism for eliminating the contaminant on the contaminated zirconia.^51^ These findings align with the previous study conducted by Ozdemir et al, who demonstrated that universal cleaning agent can efficiently remove contaminants from translucent zirconia restorations.^42^ Silva et al identified sandblasting with Al_2_O_3_ demonstrated superior bond strength compared to the cleaning solution (IC).^21^ Both methods also resulted in higher resin bond strength to zirconia compared to water cleaning.^50^ The alkaline cleaning solution (IC) is also used in this study to clean artificial saliva contaminants after sandblasting. Unlike previous studies,^3,20,24,63,64^ sandblasting was used for surface cleaning after contamination, not before saliva contamination. Significantly higher bond strength values were observed in the sandblasting and IC group combination than in the only sandblasted and IC-applied groups. This finding can be related to the contributing effect of sandblasting that may have created active micro retentive zirconia surfaces that can bond with the resin cement containing phosphate monomers.^64^

Ivoclean solution also binds to phosphate contaminants actively present on this sandblasted zirconia surface.^21^ While numerous studies^55,59^ have demonstrated fast adhesion strength between zirconia and resin following different surface treatments, all samples were kept in water at 37°C for 24 h before the tensile bond strength test. The thermal cycle was applied to the samples before the bond strength test to imitate the clinical circumstances in the oral environment. Studies have demonstrated a notable reduction in the load required to cause fractures in specimens undergoing artificial aging procedures.^16,17^ This study utilized 5,000 cycles in a thermocycling device, which is believed to simulate six months of in vivo usage, and 10,000 preload cycles.^16^ Before cementation, thermocycling was conducted, which may be subject to debate as it has primarily been contended to impact the cement.^38^

Self-adhesive resins are preferred in the study due to the advantage of accelerating the clinical procedure and eliminating the extra adhesive steps.^13^ Some reports have shown that using primer or resins with 4-META or (MDP) results in a better adhesion strength because they maintain chemical adhesion with zirconia.^8^ On the other hand, self-adhesive resin cements are more hydrophilic than other cements due to the acidic phosphate functional monomers they contain, show higher water absorption, and are more prone to hydrolytic degradation.^12,47,58^ Although this may weaken the bond strength between the resin cement and the monolithic zirconia ceramic, the bond strengths of the study after thermal cycle tests were found to be entirely satisfactory.

Various test methods such as macro shear, micro shear, macro tensile, and micro tensile tests are recommended to evaluate the bond strength of resin-based materials to dental ceramics.^61^ There is no consensus in the dental literature about which method is more effective for testing adequate adhesion between resin cement and dental ceramics. However, shear bond strength tests can be beneficial for faster sorting of materials and systems.^18^

All samples in the sandblasting group, the sandblasting+ IC group, and the IC group exhibited a mixed failure type in the examined stereomicroscope and SEM images. Shear bond strength was found to be associated with the mode of failure in each group. The results showed a mixed failure, indicating a strong bond between zirconia and adhesive resin that was not affected by contamination from saliva.^57^ In the air-water, control, pumice, and alcohol groups, most failure types were mixed, but adhesive failures were also observed. These results are consistent with the bond strength values obtained in previous studies.^3,18^ The mixed failure type seen in the samples shows that the adhesion between the zirconia and the adhesive resin is higher than the cohesive strength of the adhesive resin itself.^57^ The adhesive failure type, which is seen with decreasing bond strength values, shows that the contaminants are not removed from the zirconia surface. Saliva contaminants prevent the chemical bonding between the adhesive resin and zirconia, and the connection interface is further destroyed during thermal cycling.^66^

AES and X-ray photoelectron spectroscopy (XPS) are proximity techniques with a maximum detection depth of approximately 10 nm.^57^ On the other hand, EDS is a technique sensitive to volume changes. The study included this sample to ascertain the sub-surface’s elemental composition rather than limiting the investigation to just the outer atomic layers.^57^ Wattasirmkit et al^57^ conducted an EDS analysis on a Y-TZP dental ceramic that was treated with CoJetTM Sand. EDS was employed to conduct elemental analysis of the surfaces in all groups. In theory, carbon should be present in the contaminated specimens because carbon is a component of proteins found in saliva. Nevertheless, the EDS investigation revealed the presence of carbon in all groups, including the non-contaminated group.^57^ According to the EDS analysis results, the absence of C atoms in uncontaminated specimens and their presence after contamination with artificial saliva indicates that the organic components of the saliva could not be completely removed with the cleaning methods applied to the zirconia surface.^63^ While no C content was observed in the uncontaminated samples in this study, an organic layer consisting of C, O, and Si was observed on the ceramic surface after saliva contamination. However, Zr, Al, and O were not suitable elements for investigating contamination, as they are already present in the main composition of monolithic zirconia ceramic.^57^ The presence of this organic layer on the surface causes a decrease in bond strength.^15^ According to the EDS results, the amount of C remaining on the surface is consistent with the bond strength results. The lowest C ratio was seen in the group treated with IC, followed by sandblasting and sandblasting + IC-applied groups. The highest C ratio was in the alcohol-applied group. IC application has increased the bond strength by removing the C atoms on the surface. Although the C ratio of the sandblasted groups is higher than that of the IC group, the high bond strength may be due to the rough surface caused by sandblasting, which increased the micromechanical connection.^26^ Organic and inorganic contaminants can easily adhere to micropores caused by sandblasting. Removing contaminants from surfaces with complex surface textures is more difficult than from smooth surfaces. In this study, saliva contaminants could not be completely removed from the micropores in the sandblasting + IC group, but the remaining surface texture was sufficient for micromechanical retention. This result is consistent with Phark et al’s previous study.^43^

One of the limitations of this study is that the effect of the applied surface treatments on the surface roughness values is not examined. The other limitation is that the EDS analysis did not show the exact amount of the element on the studied surface. These results should be supported by XPS, a highly sensitive technique used to determine the chemical composition of polyphase components.^37^ The findings of this study should be supported by studies in which human saliva is used, and more research is needed to clarify whether cleaning solutions are superior to sandblasting in terms of long-term clinical bond strength.

## CONCLUSIONS

Universal cleaning agents are helpful in decontaminating saliva-contaminated zirconia during the intraoral try-in stage to recover and improve the original bond strength of cementation. Applying universal cleansing agents can be easier, less time-consuming, and more environmentally friendly than sandblasting to remove contaminants on restorations with complex surface geometry.

When Ivoclean is additionally applied after sandblasting, a strong bond to zirconia can be obtained by using primer and self-adhesive resin cement. Additionally, the potential drawbacks of additional sandblasting can be avoided in clinical practice.

Using pumice, water, and isopropyl alcohol to clean dental zirconia contaminated with saliva was not effective in fully restoring the bond strength. While pumice application resulted in slightly higher bond strength values compared to air-water, control, and isopropyl alcohol groups, the difference was not significant.

The results of the EDS analysis support the idea that the sandblasted surfaces make it easier for organic contaminants to accumulate. This result also suggests that universal cleaning agents may be preferred after sandblasting.
